# Risk of surgical site infection and efficacy of antibiotic prophylaxis: a cohort study of appendectomy patients in Thailand

**DOI:** 10.1186/1471-2334-6-111

**Published:** 2006-07-12

**Authors:** Nongyao Kasatpibal, Mette Nørgaard, Henrik Toft Sørensen, Henrik Carl Schønheyder, Silom Jamulitrat, Virasakdi Chongsuvivatwong

**Affiliations:** 1 Department of Clinical Epidemiology, Aarhus University Hospital, Aalborg and Aarhus, Denmark; 2 Department of Fundamental Nursing, Faculty of Nursing, Chiang Mai University, Chiang Mai, Thailand; 3 Department of Clinical Microbiology, Aalborg Hospital, Aarhus University Hospital, Aalborg, Denmark; 4 Department of Community Medicine, Faculty of Medicine, Prince of Songkla University, Hat Yai, Songkhla, Thailand; 5 Epidemiology Unit, Faculty of Medicine, Prince of Songkla University, Hat Yai, Songkhla, Thailand

## Abstract

**Background:**

No data currently exist about use of antibiotics to prevent surgical site infections (SSI) among patients undergoing appendectomy in Thailand. We therefore examined risk factors, use, and efficacy of prophylactic antibiotics for surgical site infection SSI among patients with uncomplicated open appendectomy.

**Methods:**

From July 1, 2003 to June 30, 2004 we conducted a prospective cohort study in eight hospitals in Thailand. We used the National Nosocomial Infection Surveillance (NNIS) system criteria to identify SSI associated with appendectomy. We used logistic regression analysis to obtain relative risk estimates for predictors of SSI.

**Results:**

Among 2139 appendectomy patients, we identified 26 SSIs, yielding a SSI rate of 1.2 infections/100 operations. Ninety-two percent of all patients (95% CI, 91.0–93.3) received antibiotic prophylaxis. Metronidazole and gentamicin were the two most common antibiotic agents, with a combined single dose administered in 39% of cases. In 54% of cases, antibiotic prophylaxis was administered for one day. We found that a prolonged duration of operation was significantly associated with an increased SSI risk. Antibiotic prophylaxis was significantly associated with a decreased risk of SSI regardless of whether the antibiotic was administered preoperatively or intraoperatively. Compared with no antibiotic prophylaxis, SSI relative risks for combined single-dose of metronidazole and gentamicin, one-day prophylaxis, and multiple-day antibiotic prophylaxis were 0.28 (0.09–0.90), 0.30 (0.11–0.88) and 0.32 (0.10–0.98), respectively.

**Conclusion:**

Single-dose combination of metronidazole and gentamicin seems sufficient to reduce SSIs in uncomplicated appendicitis patients despite whether the antibiotic was administered preoperatively or intraoperatively.

## Background

Data regarding risk factors and use of antibiotics in surgical patients are essential for preventing and treating surgical site infections (SSI). Appendectomy is one of the most common surgical procedures [1] with SSI complicating 1–5% of appendectomy cases [2-4]. One established risk factor for SSI in appendectomy is the duration of operation [1].

While antibiotic prophylaxis is common in surgical procedures [5], inappropriate use of antibiotics occurs in 25–50% of general elective surgeries [6-10]. The efficacy of antibiotic prophylaxis in patients undergoing appendectomy has been examined in several randomized and observational studies [4,11-19] showing that appropriate use of antibiotics reduces the risk of SSI following appendectomy by 40–60%.

In Thailand, some hospitals had their own internal antibiotic prophylaxis guidelines. However, standardized national guidelines for antibiotic prophylaxis among appendectomy patients have not yet been established. Simple appendicitis is treated according to surgeons' discretion, which results in use of many different agents.

No study has been conducted of the efficacy of antibiotic prophylaxis on risk of SSI in patients undergoing appendectomy in Thailand. We aimed to examine risk factors for SSI, the use of antibiotic prophylaxis, and the efficacy of antibiotic prophylaxis in reducing SSI among appendectomy patients in Thailand.

## Methods

This prospective multicenter cohort study was conducted from 1 July 2003 to 30 June 2004 in eight Thai hospitals (four tertiary care teaching hospitals and four geographically dispersed general hospitals). The participating hospitals were Chiangkham Hospital, Saraburi Hospital, Bhumibol Adulyadej Hospital, Vachira Phuket Hospital, Naradhiwas Rajanagarindra Hospital, Rayong Hospital, Chumphon Khet Udomsakdi Hospital, and Udonthani Hospital. The project was approved by the Ethical Review Committee for Research in Human Subjects, the Thai Ministry of Public Health, and the Ethical Committee and/or the directors of the participating hospitals. After attending a one-day training session on data collection and diagnostic criteria, infection control nurses in each hospital prospectively collected and recorded data.

Operating room (OR) logbooks were reviewed daily to identify uncomplicated open appendectomies meeting the inclusion criteria. We excluded appendectomies incidental to other operative procedures and the patients who were on antibiotic therapy. Patients' names, hospital numbers, and wards were identified via OR records. Medical records, operative notes, anesthetic records, diagnostic imaging reports, microbiological and biochemical data, and data on the operative procedure (duration and type of operation) were reviewed by study nurses and attending physicians. The American Society of Anesthesiologists (ASA) score of patient physical status was abstracted from anesthetic records. Data on the use of antibiotic prophylaxis included timing of first antibiotic prophylaxis dose, antibiotic agent, and duration of antibiotic therapy. These were obtained from patients' medical and anesthetic records. Following review, pertinent data were recorded on preprinted data collection forms.

Outpatient records of discharged patients and medical records of readmitted patients were also reviewed for evidence of infections developing after hospital discharge. Completed data collection forms were edited and analyzed at the study data processing center.

### Definitions

We used criteria of the US Centers for Disease Control and Prevention (CDC) NNIS System to diagnose SSI. Infections were classified as superficial incisional, deep incisional, or organ/space SSI [20]. The ASA score was used to characterize the patients' physical status as 1 (healthy), 2 (mild systemic disease), 3 (severe systemic disease), 4 (severe life-threatening systemic disease), or 5 (moribund) [21]. Patients' final diagnoses and operations were coded according to the International Classification of Diseases 10th Revision (ICD-10) and the International Classification of Diseases 9th Revision, Clinical Modification (ICD-9 CM), respectively. The appendectomy procedures were also classified according to the NNIS [22].

We defined uncomplicated appendicitis as acutely inflamed appendicitis without perforation (clean-contaminated wound). Patients with gangrenous appendicitis, peritonitis, or abscess formation were not included.

### Statistical analysis

The rate of SSI was computed by dividing the number of infections by the number of operations performed and multiplying by one hundred.

Contingency tables were constructed to analyze the relations between SSI and the other study variables: use and duration of antibiotic prophylaxis, sex, age, length of preoperative stay, type of operation, ASA score, and duration of operation. We conducted logistic regression analysis to estimate the relative risk (RR) of SSI for the main study variables.

All analyses were performed using STATA statistical software, version 7 (Stata Corp, College Station, TX).

## Results

### Patient and operation characteristics

During the study period, 2139 patients, 53.1% of them women, underwent open appendectomy. The median age was 26 years (interquartile range 16 to 39). Twenty-one percent of patients were hospitalized preoperatively with a median length of preoperative stay of 1 day (interquartile range 1 to 1). For postoperative or total hospital stay, median length was 3 days (interquartile range 2 to 4). Among 2139 operations, 72.4% were classified as emergency. The median operation duration was 58 minutes (interquartile range 42 to 83).

### SSI rates

Twenty-six SSIs were identified in 2139 operations, yielding an overall SSI rate of 1.2 infections/100 operations. Superficial and deep incisional SSIs occurred most frequently (46.2% each). Of the 26 SSIs, 15 (57.7%) were detected after hospital discharge, and a half, within seven days after surgery. The median onset of SSI was 8 days (interquartile range 5–11).

### Risk factors

The following variables were associated with the risk of SSI in the crude analyses: duration of antibiotic prophylaxis, age, elevated ASA score, prolonged preoperative hospital stay, duration of operation, emergency surgery, and sex. However, after adjustment, only prolonged duration of operation remained significantly associated with an increased risk of SSI (RR = 3.29; 95% CI 1.44–7.52) (Table 1).

**Table 1:** The association between selected risk factors and surgical site infections

**Risk factors**	**N**	**Infection**	Rate*	**Relative risk**			
				
				**Crude**	**95% CI**	Adjusted**	**95% CI**
Duration of antibiotic prophylaxis							
None	167	5	3.0	1.00	Reference	1.00	Reference
1 day	1159	12	1.0	0.34	0.12–0.97	0.30	0.10–0.89
>1 day	813	9	1.1	0.36	0.12–1.09	0.29	0.09–0.92
Duration of operation							
≤ 1.0 hour	1738	16	0.9	1.00	Reference	1.00	Reference
>1.0 hour	401	10	2.5	2.75	1.24–6.11	3.29	1.44–7.52
Age							
1–20 years	808	6	0.7	1.00	Reference	1.00	Reference
21–40 years	853	14	1.6	2.23	0.85–5.83	2.15	0.82–5.68
41–60 years	382	5	1.3	1.77	0.54–5.85	1.91	0.57–6.48
>60 years	96	1	1.0	1.41	0.17–11.81	2.11	0.22–20.76
Type of operation							
Elective	591	9	1.5	1.00	Reference	1.00	Reference
Emergency	1548	17	1.1	0.72	0.32–1.62	0.78	0.33–1.83

**Total**	**2139**	**26**	**1.2**	**-**	**-**	**-**	**-**

* Rate = # infections/100 operations

**Adjusted for sex, age, length of preoperative stay, type of operation, ASA score, and duration of operation

### The use of antibiotic prophylaxis

Prophylactic antibiotics were administered in the course of 1972/2139 (92.2%) operations. In 89.8% of these cases, antibiotics were given preoperatively, but they were given within one hour before the incision in only 38.9% of cases. The most common prophylactic antibiotics were metronidazole and gentamicin (64.2%). The combination of a single dose of metronidazole and gentamicin was used in 38.8% of cases. Antibiotic prophylaxis was administered for one day in 54.2% of cases. The median duration of antibiotic prophylaxis was 1 day (interquartile range 1 to 2). Antibiotic prophylaxis was extended for over one day in 38.0% of patients (Table 2).

**Table 2:** Characteristics of antibiotic prophylaxis administration

**Characteristics**	**Number**	**%**	**Infection**	Rate*
Antibiotic prophylaxis (N = 2139)				
No	167	7.8	5	3.0
Yes	1972	92.2	21	1.1
Time of first antibiotic dose administration (N = 1972)				
>1 hour preoperatively	1004	50.9	8	0.8
≤ 1 hour preoperatively	767	38.9	9	1.2
Intraoperatively	47	2.4	0	0.0
Postoperatively	154	7.8	4	2.6
Antibiotic agent (N = 1972)				
Combination Metronidazole and Gentamicin	1266	64.2	13	1.0
Single dose combination	766	38.8	8	1.0
Combination within 1 day	130	6.6	1	0.8
Combination > 1 day	370	18.8	4	1.1
Others**	706	35.8	8	1.1
Single dose	108	5.5	2	1.9
Multiple dose or combination within 1 day	155	7.9	1	0.6
Combination > 1 day	443	22.4	5	1.1
Duration of antibiotic prophylaxis*** (N = 2139)				
None	167	7.8	5	3.0
1 day	1159	54.2	12	1.0
>1 day	813	38.0	9	1.1

* Rate = infections/100 operations

** Others: Penicillin, amoxicillin, amoxicillin/clavulanate, ampicillin, cloxacillin, cefazolin, cephalexin, cefotaxime, ceftriaxone, ceftazidime, cefdinir, cefoxitin, ofloxacin, norfloxacin, cotrimoxazole, chloramphenical, fosfomycin, clindamycin, and amikacin.

*** Median = 1 day (interquartile range 1–2 days)

The doses of antibiotic prophylaxis administrated in this study were metronidazole 500 mg, gentamicin 80 mg, penicillin 2 million units, amoxicillin 500 mg, amoxicillin/clavulanate 1.2 g, ampicillin 1 g, cloxacillin 1 g, cefazolin 1 g, cephalexin 1 g, cefotaxime 1 g, ceftriaxone 2 g, ceftazidime 1 g, cefdinir 100 mg, cefoxitin 1 g, ofloxacin 200 mg, norfloxacin 400 mg, cotrimoxazole 480 mg, chloramphenical 1 g, fosfomycin 1 g, clindamycin 600 mg, and amikacin 500 mg.

### Efficacy of antibiotic prophylaxis

Antibiotic prophylaxis was associated with decreased risk of SSI, while timing of administration – preoperatively vs. intraoperatively – had no effect on risk. Compared with no antibiotic prophylaxis, receiving one-day, or multiple-day antibiotic prophylaxis was each associated with about one third of the SSI risk, adjusted RR were 0.30 (0.11–0.88) and 0.32 (0.10–0.98), respectively (Table 3). This reduced risk was also found when receiving only a single dose of metronidazole in combination with gentamycin (adjusted RR = 0.28 (0.09–0.90)), Data on duration, administration time, and antibiotic agent associated with SSI, adjusted for age, sex, ASA score, and duration of operation, are shown in Table 3.

**Table 3:** Association between surgical site infections and duration, timing, and antibiotic prophylaxis agent, adjusted for age, sex, ASA score, and duration of operation

**Predictor variable**	**N**	**Infection**	Rate*	**Relative risk**			
				
				**Crude**	**95% CI**	Adjusted**	**95% CI**
Duration of antibiotic prophylaxis							
None	167	5	3.0	1.00	Reference	1.00	Reference
1 day	1159	12	1.0	0.34	0.12–0.97	0.30	0.10–0.88
>1 day	813	9	1.1	0.36	0.12–1.09	0.32	0.10–0.98
Time of first antibiotic dose administration							
None	167	5	3.0	1.00	Reference	1.00	Reference
>1 hour preoperatively	1004	8	0.8	0.26	0.08–0.81	0.22	0.07–0.70
≤ 1 hour preoperatively or interoperation	814	9	1.1	0.36	0.12–1.09	0.33	0.11–1.02
Postoperatively	154	4	2.6	0.86	0.23–3.28	0.78	0.20–3.00
Antibiotic agent							
None	167	5	3.0	1.00	Reference	1.00	Reference
Single dose combination metronidazole and gentamicin	766	8	1.0	0.34	0.11–1.06	0.28	0.09–0.90
Combination metronidazole and gentamicin within 1 day	130	1	0.8	0.25	0.03–2.18	0.22	0.03–1.98
Combination metronidazole and gentamicin > 1 day	370	4	1.1	0.35	0.09–1.34	0.29	0.08–1.12
Others***	706	8	1.1	0.37	0.12–1.15	0.37	0.12–1.15

* Rate = infections/100 operations

** Adjusted for age, sex, ASA score, and duration of operation

*** Others: Penicillin, amoxicillin, amoxicillin/clavulanate, ampicillin, cloxacillin, cefazolin, cephalexin, cefotaxime, ceftriaxone, ceftazidime, cefdinir, cefoxitin, ofloxacin, norfloxacin, cotrimoxazole, chloramphenical, fosfomycin, clindamycin, and amikacin.

## Discussion

This prospective cohort study conducted in eight Thai hospitals showed that a prolonged duration of operation was a significant risk factor for SSI among patients undergoing appendectomy. Conversely, antibiotic prophylaxis was inversely related to the risk of SSI in such uncomplicated appendicitis patients. Single-day antibiotic prophylaxis was found to be as effective as multiple-day antibiotic prophylaxis in reducing SSIs and administering prophylaxis before the incision or intraoperatively did not affect the risk. A combined single dose of metronidazole and gentamicin seemed sufficient to reduce risk of SSI in uncomplicated appendicitis patients.

### Study strengths and weaknesses

The strengths of this study are its large sample size and prospective cohort design with complete early follow-up. Furthermore, we were able to include all patients admitted with uncomplicated appendicitis, who underwent open surgery in all of the eight hospitals.

Among the study limitations was the failure to account for differences in surgical technique, preoperative and postoperative practices. However, appendectomies are performed quite uniformly in Thailand and we consider large variation in these practices unlikely. Owing to high cost of post-discharge surveillance, we were unable to follow all patients for 30 days after surgery. Curtailed follow-up may cause underestimation of the SSI rate. Still, the SSI rate in our study (1.2%) was similar to that observed in the NNIS system report (1.3%) [2]. Some SSIs were potentially misclassified when the exact layer of tissue or organ/space involved in the infection was unclear. To limit such misclassification, an expert was consulted in these cases. In addition, the surgeon and at least one person from the infection control team had to agree to the diagnosis and classification of SSI in all cases.

### Risk factors

A prolonged duration of operation has been reported as a risk factor for SSI in other studies [1,23,24]. Earlier investigations have also reported increasing age [25], and emergency surgery [26] as risk factors of SSI. Our study did not show substantial association between these factors and the risk of SSI. Under-accounting for other predictors of SSI not included in our analysis might explain our failure to observe an association between age or emergency surgery and SSI.

### The use of antibiotic prophylaxis

Twenty-one different antibiotic agents were administered to appendectomy patients in our study, highlighting the lack of consensus among Thai surgeons in prescribing practices. Metronidazole plus gentamicin were most commonly used agents, and our study confirmed their effectiveness in reducing SSIs reported by others [18,19]. The prophylaxis was effective despite the fact that timing of prophylaxis followed international guidelines in only 39% of the cases [27,28]. This finding corroborates the notion that the timing of the administration – pre-, intra- or post-operation – may not be crucial for preventing SSIs [1]. At the same time, higher SSI rate was reported among patients who received antibiotic prophylaxis after surgical incision [29], probably because the antibiotic serum concentration at the surgical closure is strongly associated with SSI [30].

The American Society of Health System Pharmacists (ASHP) [27] recommends prophylaxis with cephalosporins for uncomplicated appendicitis [27,28], with metronidazole and gentamicin only considered an alternative in cases of penicillin allergy. However, the combination of metronidazole plus gentamicin may have an economic advantage. A Thai study indicated that the estimated cost for these combined agents was 210 baht per 24 hours, compared with 1160 baht for cefoxitin [31]. Adverse effects were not documented for our patients.

Extended duration of antibiotic prophylaxis was less frequent in our study than in a Malaysian study [32], but our finding is consistent with other studies conducted in France [8] and Spain [9].

The improper use of antibiotic agents and inappropriately prolonged duration of antibiotic prophylaxis are likely to cause antimicrobial resistance [33-38]. Surgeons and surgical departments need to update their practices of antibiotic prophylaxis to comply with standard guidelines [27,28] and updated evidencebase [1].

### Efficacy of antibiotic prophylaxis

Antibiotic prophylaxis is associated with a decreased risk of postoperative SSIs. This finding is in agreement with other studies [4,14-19]. In addition, our data indicate that in uncomplicated appendicitis cases, one-day antibiotic prophylaxis is just as effective in reducing SSIs as multiple-day antibiotic prophylaxis. Thus, our study does not offer justification for routine administration of oral antibiotics upon hospital discharge as in a previous study [39]. Furthermore, our findings correspond to findings in other reports [27,40], a single-dose antibiotic [40], such a combined a single dose of metronidazole and gentamicin, was efficient to reduce SSIs in uncomplicated appendicitis [27].

## Conclusion

A prolonged duration of operation was associated with increased risk of SSI among appendectomy patients, while antibiotic prophylaxis was associated with decreased risk. A combined a single dose of metronidazole and gentamicin administered preoperatively or intraoperatively appears sufficient to reduce SSIs in patients with uncomplicated appendicitis. We recommend that preoperative antibiotic prophylaxis be administered to all patients undergoing appendectomy.

## Abbreviations

SSI Surgical site infection

NNIS National Nosocomial Infection Surveillance

ASA American Society of Anesthesiologists

RR Relative risk

CI Confidence intervals

## Competing interests

The author(s) declare that they have no competing interests.

## Authors' contributions

HTS, NK and MN conceptualized the study. NK and the Surgical Site Infection Study Group assisted with the data collection. NK was responsible for data management and data analysis. HTS, NK, MN and HCS were responsible for interpretation of data. HTS, MN, HCS, SJ and VC provided advice and review. NK, HTS, MN, HCS, SJ and VC collaboratively wrote the manuscript. All authors read and approved the final manuscript.

## Pre-publication history

The pre-publication history for this paper can be accessed here:



## References

[B1] Andersen BR, Kallehave FL, Andersen HK (2005). Antibiotics versus placebo for prevention of postoperative infection after appendicectomy. Cochrane Database Syst Rev.

[B2] (2004). National Nosocomial Infections Surveillance (NNIS) System Report, data summary from January 1992 through June 2004, issued October 2004. Am J Infect Control.

[B3] Hale DA, Molloy M, Pearl RH, Schutt DC, Jaques DP (1997). Appendectomy: a contemporary appraisal. Ann Surg.

[B4] Koch A, Zippel R, Marusch F, Schmidt U, Gastinger I, Lippert H (2000). Prospective multicenter study of antibiotic prophylaxis in operative treatment of appendicitis. Dig Surg.

[B5] Dellinger EP, Gross PA, Barrett TL, Krause PJ, Martone WJ, McGowan JE, Sweet RL, Wenzel RP (1994). Quality standard for antimicrobial prophylaxis in surgical procedures. Infectious Diseases Society of America. Clin Infect Dis.

[B6] Gyssens IC, Geerligs IE, Nannini-Bergman MG, Knape JT, Hekster YA, van der Meer JW (1996). Optimizing the timing of antimicrobial prophylaxis in surgery: an intervention study. J Antimicrob Chemother.

[B7] Silver A, Eichorn A, Kral J, Pickett G, Barie P, Pryor V, Dearie MB (1996). Timeliness and use of antibiotic prophylaxis in selected inpatient surgical procedures. The Antibiotic Prophylaxis Study Group. Am J Surg.

[B8] Bedouch P, Labarere J, Chirpaz E, Allenet B, Lepape A, Fourny M, Pavese P, Girardet P, Merloz P, Saragaglia D, Calop J, Francois P (2004). Compliance with guidelines on antibiotic prophylaxis in total hip replacement surgery: results of a retrospective study of 416 patients in a teaching hospital. Infect Control Hosp Epidemiol.

[B9] Pons-Busom M, Aguas-Compaired M, Delas J, Eguileor-Partearroyo B (2004). Compliance with local guidelines for antibiotic prophylaxis in surgery. Infect Control Hosp Epidemiol.

[B10] Dahms RA, Johnson EM, Statz CL, Lee JT, Dunn DL, Beilman GJ (1998). Third-generation cephalosporins and vancomycin as risk factors for postoperative vancomycin-resistant enterococcus infection. Arch Surg.

[B11] Lau WY, Fan ST, Chu KW, Suen HC, Yiu TF, Wong KK (1985). Randomized, prospective, and double-blind trial of new beta-lactams in the treatment of appendicitis. Antimicrob Agents Chemother.

[B12] Liberman MA, Greason KL, Frame S, Ragland JJ (1995). Single-dose cefotetan or cefoxitin versus multiple-dose cefoxitin as prophylaxis in patients undergoing appendectomy for acute nonperforated appendicitis. J Am Coll Surg.

[B13] Soderquist-Elinder C, Hirsch K, Bergdahl S, Rutqvist J, Frenckner B (1995). Prophylactic antibiotics in uncomplicated appendicitis during childhood – a prospective randomised study. Eur J Pediatr Surg.

[B14] Nguyen BL, Raynor S, Thompson JS (1992). Selective versus routine antibiotic use in acute appendicitis. Am Surg.

[B15] Gorecki P, Schein M, Rucinski JC, Wise L (1999). Antibiotic administration in patients undergoing common surgical procedures in a community teaching hospital: the chaos continues. World J Surg.

[B16] Tonz M, Schmid P, Kaiser G (2000). Antibiotic prophylaxis for appendectomy in children: critical appraisal. World J Surg.

[B17] Charalambous C, Tryfonidis M, Swindell R, Lipsett AP (2003). When should old therapies be abandoned? A modern look at old studies on topical ampicillin. J Infect.

[B18] Lau WY, Fan ST, Chu KW, Yip WC, Yiu TF, Yeung C, Wong KK (1986). Cefoxitin versus gentamicin and metronidazole in prevention of post-appendicectomy sepsis: a randomized, prospective trial. J Antimicrob Chemother.

[B19] al Dhohayan A, al Sebayl M, Shibl A, al Eshalwy S, Kattan K, al Saleh M (1993). Comparative study of augmentin versus metronidazole/gentamicin in the prevention of infections after appendicectomy. Eur Surg Res.

[B20] Horan TC, Gaynes RP, Martone WJ, Jarvis WR, Emori TG (1992). CDC definitions of nosocomial surgical site infections, 1992: a modification of CDC definitions of surgical wound infections. Am J Infect Control.

[B21] Owens WD, Felts JA, Spitznagel EL (1978). ASA physical status classifications: a study of consistency of ratings. Anesthesiology.

[B22] Culver DH, Horan TC, Gaynes RP, Martone WJ, Jarvis WR, Emori TG, Banerjee SN, Edwards JR, Tolson JS, Henderson TS, Hughes JM, the National Nosocomial Infections Surveillance System, Atlanta, Georgia (1991). Surgical wound infection rates by wound class, operative procedure, and patient risk index. National Nosocomial Infections Surveillance System. Am J Med.

[B23] Razavi SM, Ibrahimpoor M, Sabouri KA, Jafarian A (2005). Abdominal surgical site infections: incidence and risk factors at an Iranian teaching hospital. BMC Surg.

[B24] Hernandez K, Ramos E, Seas C, Henostroza G, Gotuzzo E (2005). Incidence of and risk factors for surgical-site infections in a Peruvian hospital. Infect Control Hosp Epidemiol.

[B25] Kaye KS, Schmit K, Pieper C, Sloane R, Caughlan KF, Sexton DJ, Schmader KE (2005). The effect of increasing age on the risk of surgical site infection. J Infect Dis.

[B26] Sohn AH, Parvez FM, Vu T, Hai HH, Bich NN, Le Thu TA, Le Hoa TT, Thanh NH, Viet TV, Archibald LK, Banerjee SN, Jarvis WR (2002). Prevalence of surgical-site infections and patterns of antimicrobial use in a large tertiary-care hospital in Ho Chi Minh City, Vietnam. Infect Control Hosp Epidemiol.

[B27] ASHP Therapeutic Guidelines on Antimicrobial Prophylaxis in Surgery (1999). American Society of Health-System Pharmacists. Am J Health Syst Pharm.

[B28] Mangram AJ, Horan TC, Pearson ML, Silver LC, Jarvis WR (1999). Guideline for Prevention of Surgical Site Infection, 1999. Centers for Disease Control and Prevention (CDC) Hospital Infection Control Practices Advisory Committee. Am J Infect Control.

[B29] Classen DC, Evans RS, Pestotnik SL, Horn SD, Menlove RL, Burke JP (1992). The timing of prophylactic administration of antibiotics and the risk of surgical-wound infection. N Engl J Med.

[B30] Zelenitsky SA, Ariano RE, Harding GK, Silverman RE (2002). Antibiotic pharmacodynamics in surgical prophylaxis: an association between intraoperative antibiotic concentrations and efficacy. Antimicrob Agents Chemother.

[B31] Sunpaweravong S (1999). Practice guidelines for prophylactic antibiotic. Songkla Med J.

[B32] Gul YA, Hong LC, Prasannan S (2005). Appropriate antibiotic administration in elective surgical procedures: still missing the message. Asian J Surg.

[B33] Paterson DL, Rossi F, Baquero F, Hsueh PR, Woods GL, Satishchandran V, Snyder TA, Harvey CM, Teppler H, Dinubile MJ, Chow JW (2005). In vitro susceptibilities of aerobic and facultative Gram-negative bacilli isolated from patients with intra-abdominal infections worldwide: the 2003 Study for Monitoring Antimicrobial Resistance Trends (SMART). J Antimicrob Chemother.

[B34] Kusum M, Wongwanich S, Dhiraputra C, Pongpech P, Naenna P (2004). Occurrence of extended-spectrum beta-lactamase in clinical isolates of Klebsiella pneumoniae in a University Hospital, Thailand. J Med Assoc Thai.

[B35] Girlich D, Naas T, Leelaporn A, Poirel L, Fennewald M, Nordmann P (2002). Nosocomial spread of the integron-located veb-1-like cassette encoding an extended-pectrum beta-lactamase in Pseudomonas aeruginosa in Thailand. Clin Infect Dis.

[B36] Girlich D, Poirel L, Leelaporn A, Karim A, Tribuddharat C, Fennewald M, Nordmann P (2001). Molecular epidemiology of the integron-located VEB-1 extended-spectrum beta-lactamase in nosocomial enterobacterial isolates in Bangkok, Thailand. J Clin Microbiol.

[B37] Biedenbach DJ, Johnson DM, Jones RN (1999). In vitro evaluation of cefepime and other broad-spectrum beta-lactams in eight medical centers in Thailand. The Thailand Antimicrobial Resistance Study Group. Diagn Microbiol Infect Dis.

[B38] Teng LJ, Hsueh PR, Tsai JC, Liaw SJ, Ho SW, Luh KT (2002). High incidence of cefoxitin and clindamycin resistance among anaerobes in Taiwan. Antimicrob Agents Chemother.

[B39] Taylor E, Berjis A, Bosch T, Hoehne F, Ozaeta M (2004). The efficacy of postoperative oral antibiotics in appendicitis: a randomized prospective double-blinded study. Am Surg.

[B40] Mui LM, Ng CS, Wong SK, Lam YH, Fung TM, Fok KL, Chung SS, Ng EK (2005). Optimum duration of prophylactic antibiotics in acute non-perforated appendicitis. ANZ J Surg.

